# A molecular perspective on the invasibility of the southern ocean benthos: The impact of hypoxia and temperature on gene expression in South American and Antarctic *Aequiyoldia* bivalves

**DOI:** 10.3389/fphys.2023.1083240

**Published:** 2023-02-21

**Authors:** Mariano Martínez, Marcelo González-Aravena, Christoph Held, Doris Abele

**Affiliations:** ^1^ Funktionelle Ökologie, Alfred-Wegener-Institut Helmholtz-Zentrum für Polar- und Meeresforschung, Bremerhaven, Germany; ^2^ Departamento Científico, Instituto Antártico Chileno, Punta Arenas, Chile

**Keywords:** warming, non-indigenous species, alternative oxidase, west antarctic peninsula, drake passage

## Abstract

When an organism makes a long-distance transition to a new habitat, the associated environmental change is often marked and requires physiological plasticity of larvae, juveniles, or other migrant stages. Exposing shallow-water marine bivalves (*Aequiyoldia cf. eightsii*) from southern South America (SSA) and the West Antarctic Peninsula (WAP) to changes in temperature and oxygen availability, we investigated changes in gene expression in a simulated colonization experiment of the shores of a new continent after crossing of the Drake Passage, and in a warming scenario in the WAP. Bivalves from SSA were cooled from 7°C (*in situ*) to 4°C and 2°C (future warmed WAP conditions), WAP bivalves were warmed from 1.5°C (current summer *in situ*) to 4°C (warmed WAP), gene expression patterns in response to thermal stress by itself and in combination with hypoxia were measured after 10 days. Our results confirm that molecular plasticity may play a vital role for local adaptation. Hypoxia had a greater effect on the transcriptome than temperature alone. The effect was further amplified when hypoxia and temperature acted as combined stressors. The WAP bivalves showed a remarkable ability to cope with short-term exposure to hypoxia by switching to a metabolic rate depression strategy and activating the alternative oxidation pathway, whilst the SSA population showed no comparable response. In SSA, the high prevalence of apoptosis-related differentially expressed genes especially under combined higher temperatures and hypoxia indicated that the SSA *Aequiyoldia* are operating near their physiological limits already. While the effect of temperature *per se* may not represent the single most effective barrier to Antarctic colonization by South American bivalves, the current distribution patterns as well as their resilience to future conditions can be better understood by looking at the synergistic effects of temperature in conjunction with short-term exposure to hypoxia.

## 1 Introduction

Temperature and oxygen availability are key physiological variables shaping the fitness of marine ectotherms and determine their biogeographical distributions (temperature) and the ecological niche they can locally occupy (Poloczanska et al., 2013; Deutsch et al., 2015). Rising global temperatures decrease oxygen solubility in seawater and enhance stratification, reducing downward mixing of oxygen into midwater and deep layers (Shepherd et al., 2017; Li et al., 2020). Increasing oxygen demand and organic matter turnover at higher temperatures intensify spreading of hypoxic or even anoxic zones in coastal sediments and shallow waters, limiting survival of hypoxia-sensitive species while opening new space for hypoxia/anoxia-tolerant species. Hence, global warming has the potential to alter the boundaries of current species distributions (Brown and Thatje, 2015; Spence and Tingley, 2020). One approach toward understanding the biological consequences of climate change for marine biodiversity therefore lies in the prediction of future distribution of species by experimentally determining the physiological influence of changes in temperature and oxygen levels, and analysing the molecular mechanisms that shape the responding physiological pathways. The relevance of the experimental results depends strongly on a realistic prediction of future environmental scenarios for current habitats, or the scenario expected in potential new habitats. In this study, we used the protobranch bivalve *Aequiyoldia cf. eightsii* from the West Antarctic Peninsula (WAP) and southern South America (SSA) as a model to evaluate combined effects of temperature changes and hypoxia on patterns of gene transcription. Rather than increasing the intensity of a stressor or various different stressors until a physiological response can be registered (acute stress response), this study employs a different strategy in that we let both the direction and the magnitude of the departure from normal conditions (temperature) be determined by a clearly defined and realistic scenario, namely the crossing of an organism across Drake Passage from SSA to the WAP, or *vice versa*. Contrary to studies that follow an already successful invasive species in its old and new distribution range (Tepolt and Palumbi, 2015), this major dispersal event has to our knowledge not yet happened within recent times in the marine bivalve *A. cf. eightsii*. It is beyond the scope of this paper to investigate how likely a particular dispersal event may be but what happens once it has occurred, in particular what role (if any) physiology may have played in preventing this species from successfully establishing a foothold after crossing the Drake Passage in either direction until now. In parallel, we study how a temperature increase (by itself and in combination with hypoxia) leads to stress in Antarctic bivalves, which mimics either a continued warming *in situ*, or a successful northward transition across the Drake Passage towards SSA.

Since the early 2000s, the number of Antarctic cruise liners is rapidly increasing, and with the majority of scientific and logistic activities concentrating between SSA and the WAP, the risk of introducing non-indigenous species (NIS) by passive transport onto the shelves of a rapidly warming Antarctic continent is intensifying (Chown et al., 2012; Hughes et al., 2015; McCarthy et al., 2019). In addition, reports of passive introductions of NIS in kelp rafts without direct human assistance have recently increased on the WAP (Fraser et al., 2018; Avila et al., 2020). Thus, polar species/communities are not only threatened by having to cope with the fastest warming rates on Earth (Clem et al., 2020) without the possibility of evading to colder higher latitudes, but also by the greater risk of competition for resources or trophic interactions with non-indigenous species from lower latitudes (Chan and Briski, 2017). Recently, Hughes et al. (2020) identified 13 species as presenting a high risk of invading the WAP region, of which 3 correspond to *Mytilus* bivalve molluscs (mussels). Documented invasions of terrestrial and marine organisms at the WAP are increasing, and recently the blue mussel *Mytilus cf. platensis* was added to the list as the first mussel introduced on King-George Island (WAP) (Cardenas et al., 2020), a major hub of West Antarctic tourism and logistic shipping (McCarthy et al., 2022). Regarding invasions in the opposite direction (from WAP to South America), studies based on passive dispersal simulations showed that virtual drifters released from the WAP can cross the Drake Passage but may not reach South America regularly (Schiavon et al., 2021).

*Aequiyoldia* bivalves are a conspicuous genus in the Southern Ocean inhabiting soft-substratum ecosystems of SSA and the WAP, and several Subantarctic islands (González-Wevar et al., 2019). This bivalve with a maximum shell length of about 35 mm (Nolan and Clarke, 1993) occurs in soft-sediments between the subtidal and ca. 800 m depth, with the greatest abundance at depths shallower than 100 m. The species has a flexible feeding strategy, being able to switch between deposit and filter feeding strategies and consuming phytoplankton and organic detritus present in the surface layers of sediments. During deposit feeding, the animals carry out vertical feeding migrations in the sediment, involving intensive locomotory activity. Still, *A. cf. eightsii* burrows relatively shallowly compared to other protobranch species (Davenport, 1988). In contrast to other protobranch species and many Antarctic invertebrates, the reproductive ecology of *A. cf. eightsii* exhibits continuous oogenesis, with a period of increased reproductive intensity and spawning during the austral winter, and asynchrony between females (Lau et al., 2018). Although the populations of SSA and the WAP are currently formally considered the same species, recent molecular studies based on mitochondrial (González-Wevar et al., 2019) as well as additional Single Nucleotide Polymorphisms (SNPs) and nuclear markers (Martínez, 2021) suggest that populations on either side of the Drake should be considered reproductively isolated species (cytochrome oxidase p-dist. 0.06—0.08). For this reason, in the following we will call both species *A. cf. eightsii* but emphasize that Antarctic and South American populations are likely different, but closely related species awaiting formal taxonomic recognition. Recently, Muñoz-Ramírez et al. (2020) highlighted based on genomic data the role of the Antarctic Circumpolar Current as a biogeographic barrier to larval transport between both continents, preventing or strongly reducing the potential for genetic connectivity between Antarctic and South American *Aequiyoldia* lineages. A hypothetical crossing of *Aequiyoldia* across Drake Passage is then more likely to be caused by ships as larvae in ballast water.

For *A. cf. eightsii* and many Antarctic marine ectotherms, the physiological mechanisms that protect cells during acute and chronic thermal stress, oxygen deficiency and ocean acidification (e.g., heat shock response, activities of some antioxidant enzymes, metabolic regulation, etc.) have been addressed in previous studies and are at least partly understood (Abele et al., 2001; Abele and Puntarulo, 2004; Weihe et al., 2010; Clark et al., 2013; Johnson and Hofmann, 2020). Recent studies of functional responses and gene transcription (Clark et al., 2016) revealed Antarctic *Aequiyoldia* to be tolerant in a temperature ramp experiment (1°C per 1 h), relative to six other Antarctic endemic invertebrates from different clades reaching an upper lethal temperature of 25°C. However, Peck et al. (2010) showed that this species did not change its upper lethal temperature after acclimating during 60 days to a temperature 3.5°C above annual average, which the authors interpreted as poor ability for thermal acclimation in an Antarctic stenotherm. An earlier experimental study (Abele et al., 2001) showed respiration and locomotory activity of Antarctic *A. cf. eightsii* to increase steadily in response to warming (1°C per 10 h until a max. temperature of 5°C), confirming that *A. cf. eightsii* tolerates temperatures up to 5°C (max. *in situ* temperature 2°C), with burrowing activity undiminished at these temperatures. If warmed at slower pace (1°C per 5 days until a max. temperature of 11°C), temperature tolerance was even slightly higher (7°C), although accompanied by loss of antioxidant function and tissue damage in the lipid phase (i.e., membrane damage).

**FIGURE 1:**
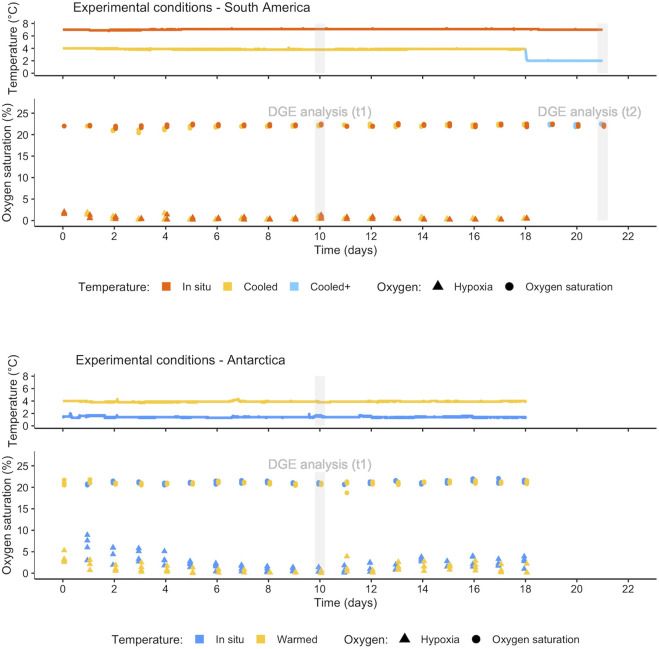
Experimental temperature and oxygen recorded during treatments in SSA and WAP: Continuous record of temperature (one measure per minute) (above) and discrete daily measures of oxygen in each replicate of the four treatments (below). Gray bars indicate the collection day (t_1_ and t_2_) for differential gene expression (DGE) analysis.

We hypothesize that the predicted warming at the WAP in combination with an increased risk of hypoxia in coastal sediments (Sampaio et al., 2021) represents a physiological stress condition for Antarctic *A. cf. eightsii*, and might imply changes in population dynamics and favour immigration across the Drake Passage from SSA to the northern WAP region. Even though both environments are predicted to warm, waters at the WAP climate are clearly colder relative to SSA, which implies that South American bivalve adults or larvae crossing the Drake would face a rather sudden cooling scenario. The experimental approach on both continents consisted in exposing bivalves to oxygen saturation (21% O_2_ saturation, from here: normoxia) and hypoxia (<2% O_2_ saturation) under current *in situ* summer temperature conditions (1.5°C in WAP and 7°C in SSA). In parallel, we tested a near future warming scenario predicted for WAP nearshore bottom water (see Morley et al., 2020) for Antarctic specimens (4°C), as well as a scenario of southward migration of SSA *Aequiyoldia* and their capacity to survive in shallow water environments of Northern WAP at present (2°C) and in the future (4°C) (Figure 1).

## 2 Materials and methods

### 2.1 Animal collection and maintenance

*Aequiyoldia cf. eightsii* for the experiments were collected in Southern South America (SSA) in October 2017 and in West Antarctic Peninsula (WAP) in January 2018. Bivalves from SSA were hand-collected by SCUBA divers in the shallow subtidal (<3 m depth) in the Rinconada Bulnes, Strait of Magellan, Chile (53°37′52″S; 70°56′54″W) on a single day in October 2017. Antarctic bivalves were collected on a single sampling day in January 2018 from one site in Potter Cove, King George Island (KGI), South Shetlands (62°14′11″S; 58°40′14″W) in 6 m water depth using a Van Veen grab (Supplementary Figure S1). At the sampling site, local *in situ* temperature and salinity were recorded with a Multiparameter HANNA (HI 9828) in SSA and with a Sea-Bird CTD (SBE19plusV2, Sea-Bird Electronics, Bellevue, WA, United States) at KGI, WAP. In addition, sediment cores were taken by SCUBA divers using cylindrical Plexiglas corer (height: 50 cm, diameter: 8 cm; 3 replicates per station) for analyses of total organic carbon (TOC) and total sulphur (TS) in the first 2 cm sediment-layer, and the ratio TOC: TS was calculated as a proxy of sediment oxygenation conditions (Togunwa and Abdullah, 2017). Physico-chemical data at the collection sites are shown in Supplementary Table S1. Experimental temperature conditions were set based on the *in situ* environmental conditions and on the existing data for both sites. The sea temperature in the coastal areas (<50 m depth) of the South Shetlands Islands (data for Potter Cove and Fildes Bay) varies annually between −2°C and 1.5°C, while in summer it varies mainly between 0.5°C and 1.5°C, reaching occasionally a maximum of 2°C. In the Strait of Magellan the annual temperature ranges from 5.9°C to 9.9°C in sublittoral waters, while in summer it ranges from 7°C to 9.9°C (Cardenas et al., 2020; Barlett et al., 2021).

At each location, bivalves were transported to the local research facility in insulated containers filled with water and sediment from the sampling site. Once in the laboratory, bivalves were immediately sorted from the muddy sediment, checked for shell damage and vitality (protruded foot in motion), and transferred to an aquarium supplied with seawater from the collection site and here maintained at *in situ* temperature. The bottom of the aquarium was covered with a 2 cm thick layer of sediment from the sampling site and allowed to settle for 2 h before adding the animals, and the aquarium was provided with aeration with the help of bubble stones and maintained in the dark during 10 days for acclimation. Incubation water was replaced with water from each sampling site every 48 h.

### 2.2 Experimental setup

After the acclimation period, four replicates of five animals each were subjected to each of the four combinations of temperature and oxygen concentration during 18 days in WAP and 21 days in SSA. The animals were randomly placed in 500 ml Kautex jars containing a 1 cm layer of sediment and water from the respective collection site. Each jar was then assigned to be exposed to treatment under one of the following experimental conditions: in WAP: *In situ* (1.5°C) and warming (4°C) in combination with normoxia (T_is_Ox_n_, T_w_Ox_n_) and hypoxia (T_is_Ox_hyp_, T_w_Ox_hyp_); in SSA: *In situ* (7°C) and South migration future scenario (4°C) in combination with normoxia (T_is_Ox_n_, T_c_Ox_n_) and hypoxia (T_is_Ox_hyp_, T_c_Ox_hyp_). The water temperature inside the jars was kept constant by submerging the jars in temperature-controlled water baths (Thermo Haake DC10-P21). Hypoxic conditions were created by providing the experimental units with a continuous water flow with 2% O_2_ saturation, with flow velocity adjusted to achieve the exchange of the total water volume of the jars (500 ml) in the course of 1 day. The water was supplied from a 10 L tank bubbled with a gas mixture 2% O_2_:98% N_2_. This design could not be replicated in the normoxic treatment, since a test run with continuous water flow at 21% O_2_ saturation through the hermetically sealed lids showed that animal and sedimentary/microbial respiration decreased oxygen to below 15% saturation within some hours, with uncontrollable variation between replicates and over time. Therefore, normoxic conditions were ensured by directly bubbling each experimental jar with air and manually replacing the total volume of water in each jar every day (without stirring up the sediment).

In both experiments, one animal per jar (i.e., four replicates per treatment) was collected at day 10 (t_1_) for differential gene expression analysis. In the experiment in SSA, an additional step was conducted on day 18, by further decreasing the temperature in the T_c_Ox_n_ treatment from 4°C down to 2°C (T_c+_Ox_n_). On day 21 (t_2_), one bivalve per jar was collected from T_is_Ox_n_ and T_c+_Ox_n_ treatments for differential gene expression analysis (Figure 1). Animals were immediately dissected on ice under a stereomicroscope after collection, and mantle tissue was conserved in RNA later (SIGMA) and stored at −80°C.

During the total period of both experiments, temperature was recorded continuously (one measure per minute) in both experimental water baths, and measurements of oxygen were performed daily in each of the four replicates in the four treatments (Figure 1). Oxygen measurements were made approximately 1 cm away from the water-sediment interface, introducing the sensor through a small gate in the lid of the jars; outside the measurement periods these openings were kept sealed. For a graphical representation of the experimental design see Supplementary Figure S2.

### 2.3 RNA extraction, library preparation and *de novo* assembly

Differential gene expression analyses were performed using an assembly created from 70 libraries (one library per individual) of *A. cf. eightsii* from SSA and WAP by Martínez (2021) as reference. In the present study, a subset of 40 libraries (out of the total 70) corresponding to the organisms of the different treatments (in SSA and WAP) were analysed for differential gene expression.

Total RNA was isolated from mantle tissue (5—30 mg) using the Direct-zol^TM^ RNA MiniPrep kit (ZYMO Research Corp., United States) according to the manufacturer’s instructions. A total of 40 individual libraries (one per specimen) were prepared using the Illumina TruSeq Stranded mRNA Sample Preparation kit starting from 1 µg of total RNA following the protocol provided by the kit. Libraries were tagged through adapter ligation, diluted to 0.8 nM, and subsequently pooled and cleaned using magnetic beads (AMPure XP, Beckmann Coulter) to remove the remaining primer content. Final cDNA concentration was measured utilizing a LabChip GX Touch (PerkinElmer, United States). The pool of samples was sequenced at the Alfred Wegener Institute on an Illumina NextSeq 500 sequencer using the NextSeq High Output Kit v2 (150 cycles) with a paired-end protocol.

Raw reads were quality controlled by FastQC v. 0.11.7 (Babraham Institute, Cambridge, UK), cleaned and *de novo* assembled using the Trinity genome-independent transcriptome assembler v2.8.4 (Grabherr et al., 2011). For details on *de novo* assembly parameters see Martínez (2021).

### 2.4 Differential gene expression and Gene Ontology analyses

The differential gene expression analysis involved the alignment of the short reads of each sample separately against the *de novo* reference transcriptome using Bowtie2 v 3.3.4.1 (Langmead et al., 2009). This involved pairwise differential gene expression analysis among the four treatments of temperature and oxygen at t_1_ in the Antarctic (WAP) experiment (*n*T_is_Ox_n_ = 4, *n*T_w_Ox_n_ = 4, *n*T_is_Ox_hyp_ = 4, *n*T_w_Ox_hyp_ = 4), and among treatments in South America (SSA) at t_1_ (*n*T_is_Ox_n_ = 4, *n*T_c_Ox_n_ = 4, *n*T_is_Ox_hyp_ = 4, *n*T_c_Ox_hyp_ = 4). Additionally, in South America, a pairwise differential gene expression analysis was carried out between normoxic treatments under *in situ* (7°C) and low (2°C) temperature exposure at t_2_ (*n*T_is_Ox_n_ = 4, *n*T_c+_Ox_n_ = 4). In order to highlight the effects of temperature further, the comparison between normoxic treatments under *in situ* (7°C) and cold exposure scenarios (4°C) at t_1_ (*n*T_is_Ox_n_ = 4, *n*T_c_Ox_n_ = 4) was also included in this analysis.

Relative abundances of transcripts were estimated by RSEM version 1.2.26 (Li and Dewey, 2011) and differential expression of genes was assessed using a test based on the negative binomial distribution as integrated in the Bioconductor R package DESeq2 (Love et al., 2014), with a significance threshold set to *p* ≤ 0.001 and a fold-change of at least 2. Gene expression results are shown graphically in a Principal Component Analysis (PCA) performed with the normalized counts per sample of every differentially expressed gene (DEG). The tools were executed using the Trinity package v 2.8.4. The annotation of the DEGs was performed using DIAMOND v 0.9.24 (Buchfink et al., 2015) including a homology search against the UniProt Swiss-Prot database and assigning Gene Ontology (GO) terms to annotated transcripts. GO enrichment analyses were carried out using GOseq (Young et al., 2010) by separately considering up- and down-regulated genes of every pairwise differential gene expression analysis and their corresponding GO annotation, and using the full list of DEGs with GO terms as the background.

## 3 Results

### 3.1 Differential gene expression analysis

In both the WAP and SSA experiments, the effect of oxygen alone (constant *in situ* temperature, 10 days) resulted in a higher number of differentially expressed genes than temperature alone (under normoxia). Moreover, at both WAP and SSA sampling sites, the highest number of DEGs clearly resulted from the comparison of T_w_Ox_hyp_ and T_is_Ox_hyp_, respectively, with the treatments of lower temperature and normoxia (Table 1). Although one experiment (WAP) involves warming and the other involves cooling (SSA; both with roughly same magnitude of change), the most notable difference between experiments from either continent was the influence of the higher experimental temperature, i.e., experimental warming of Antarctic *Aequiyoldia cf. eightsii* caused approximately five times higher number of DEGs (90 DEGs) than exposure to *in situ* temperature *vs*. cooling in South American *A. cf. eightsii* (14 DEGs, see Table 1). In other words, Antarctic *A. cf. eightsii* respond more vigorously to warming whereas hypoxia affects both populations to approximately the same extent.

**TABLE 1:** Number of differentially expressed genes (DEGs) at each pairwise comparison among the four treatments of temperature and oxygen at time t_1_ (10 days) in the experiments performed in West Antarctic Peninsula (WAP) and southern South America (SSA). In bold the highest number of DEGs at each experiment, which in both cases resulted from the comparison between higher temperature and hypoxia (T_w_Ox_hyp_ in WAP and T_is_Ox_hyp_ in SSA) and lower temperature and normoxia treatments.

	WAP					SSA			
	T_is_Ox_n_	T_is_Ox_hyp_	T_w_Ox_n_	T_w_Ox_hyp_		T_is_Ox_n_	T_is_Ox_hyp_	T_c_Ox_n_	T_c_Ox_hyp_
T_is_Ox_n_		169	90	**298**	T_is_Ox_n_		171	14	211
T_is_Ox_hyp_			93	47	T_is_Ox_hyp_			**312**	9
T_w_Ox_n_				229	T_c_Ox_n_				202
T_w_Ox_hyp_					T_c_Ox_hyp_				

**FIGURE 2:**
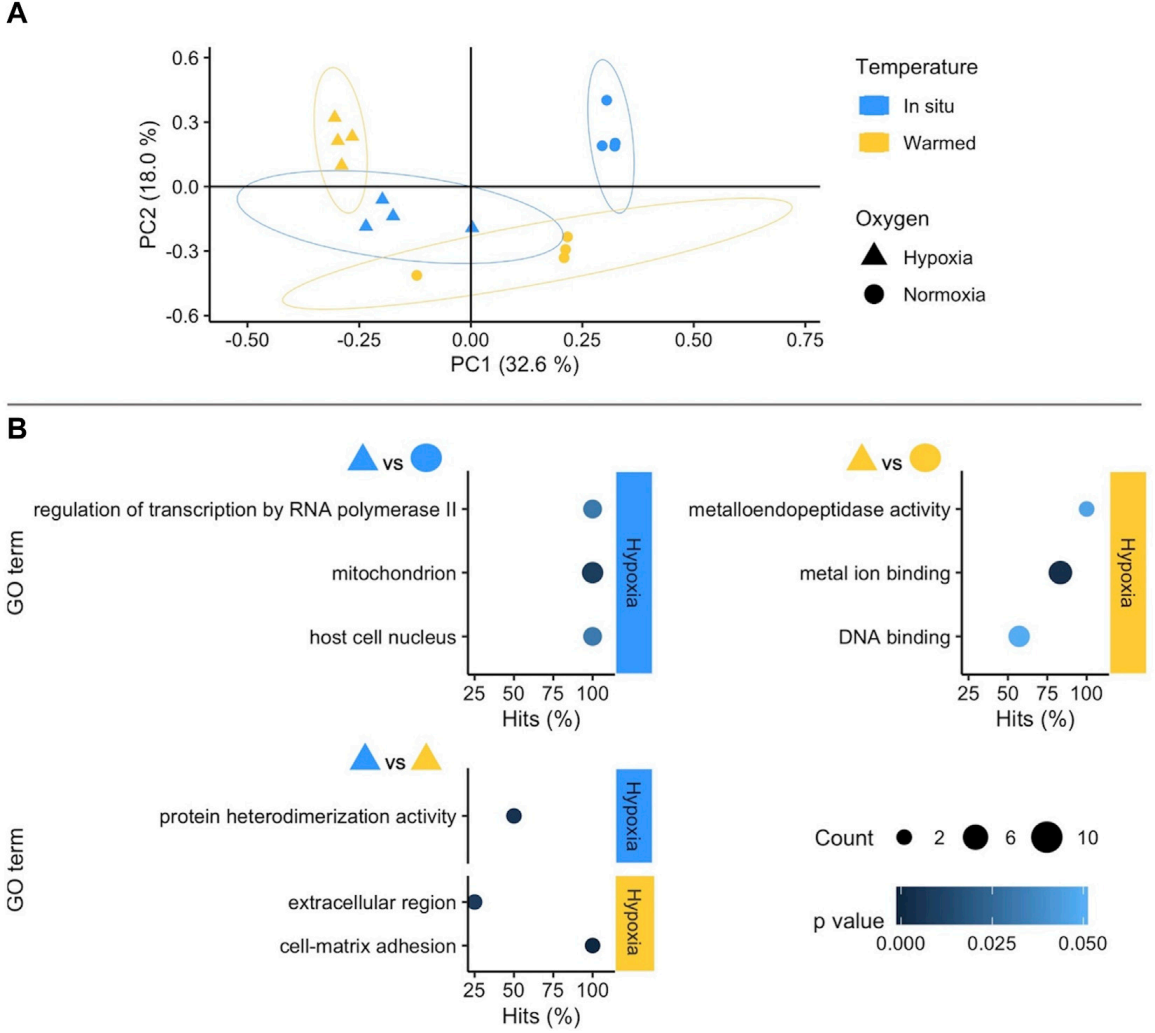
**(A)** Principal Component Analysis based on counts of differentially expressed genes (DEGs) in the West Antarctic Peninsula experiment (WAP). Colours and shapes identify temperature and oxygen treatments, respectively, and a 95% confidence ellipse is shown for each of the four treatments. **(B)** GO enrichment analysis of pairwise comparisons. Enriched terms are shown separately for the two compared treatments identifying temperature (colours) and oxygen conditions in the right side of each plot. The *x* axis indicates the percentage of DEGs found in a certain category over the total number of genes in this category (Hits). Size code indicates the number of DEGs in the GO term category (Count) and a gradient of colour the *p-value* of the over represented category. Only GO terms with more than one DEG in the category are shown in the plots (see full list in Supplementary Table S2).

**FIGURE 3:**
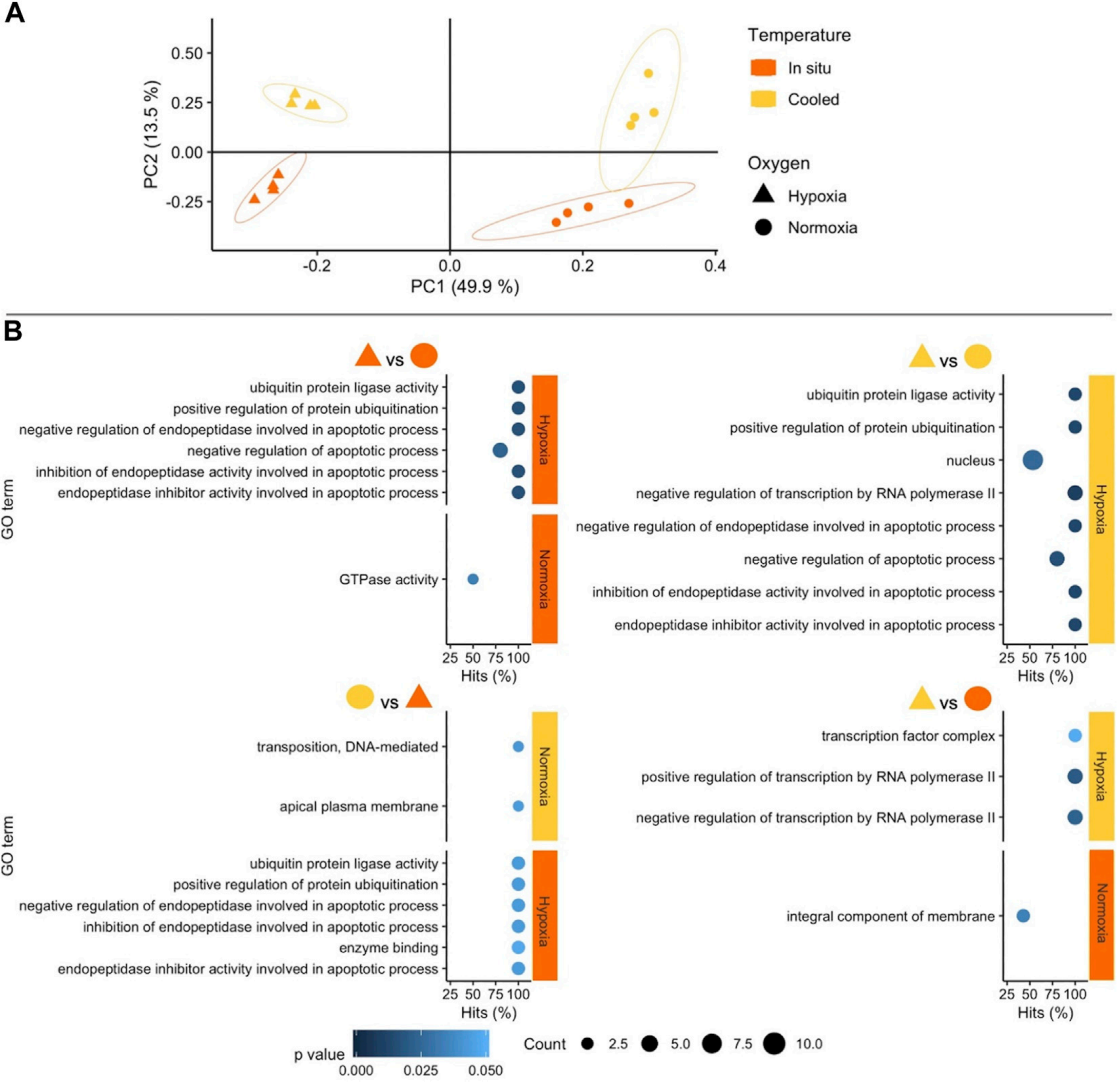
**(A)** Principal Component Analysis based on counts of differentially expressed genes (DEGs) in the experiment performed in southern South America (SSA). Colours and shapes identify temperature and oxygen treatments, respectively, and a 95% confidence ellipse is indicated for each of the four treatments. **(B)** GO enrichment analysis of pairwise comparisons. Enriched terms are shown separately for the two treatments compared identifying temperature (colours) and oxygen conditions on the right margin of each plot. The *x* axis indicates the percentage of DEGs found in each category over the total number of genes in the category (Hits). Size code indicates the number of DEGs in the GO term category (Count) and a gradient of colour the *p-value* of the over represented category. Only GO terms with more than one DEG in the category are shown in the plots (see full list in Supplementary Table S2).

The stronger influence of oxygen availability on gene expression patterns becomes obvious in the Principal Component Analysis (PCA) (Figure 2A: WAP, Figure 3A: SSA). In both PCAs, the Principal Component (PC) 1 mainly separates animals according to oxygenation conditions explaining most of the variance, whereas PC2 reflects mostly temperature conditions with a lower percentage of explained variance. This pattern is clearer in the PCA of the experiment in SSA, in which the individual response within each treatment group was more homogeneous (Figure 3A).

### 3.2 Gene ontology analysis

Oxygen availability was also the most influential factor in the enrichment of Gene Ontology (GO) terms on both continents. In SSA bivalves, the combined effects of variation in temperature and oxygen conditions also resulted in highly significant GO terms (Figure 3B). Even though temperature alone also resulted in enriched terms, these were mostly based on single DEGs and were hence treated with caution in our main analysis (but see Supplementary Table S2). It should be noted that the GO term analysis is based only on annotated genes, i.e., a subset of all DEGs, a distinction that is particularly significant here as in the study of non-model species in general.

#### 3.2.1 *Functional response to warming and hypoxia of Antarctic* Aequiyoldia

Raising the water temperature to 4°C under normoxic conditions did not elicit a clear-cut response in WAP *A. cf. eightsii*, because most GO terms were supported by single genes only. Several of these GO terms (3 out of 9, Supplementary Table S2) indicate a response of bivalve cellular immune defence.

Furthermore, the functional response to hypoxia in Antarctic *Aequiyoldia* was temperature-dependent. Under *in situ* temperature the most significant GO term was “mitochondrion” involving the upregulation of the Alternative Oxidase (AOX), an alternative mitochondrial electron transport system (ETS) component that operates when cytochrome oxidase is inhibited by nitric oxide or sulfide leading to a metabolic rate reduction; and extracellular metal binding proteins (e.g., Haloacid dehalogenase) (see Supplementary Table S3). In individuals exposed to hypoxia at higher temperature, the GO term ‘metal ion binding’ was most significant. Upregulation of (transition) metal binding capacity (in combination with the other enriched GO term “metalloendopeptidase activity”) primarily points to activation of metal chaperones (Cu, Zn, Fe) or metal storage/transport proteins such as ferritin or transferrin. This function supports safe storage and translocation of harmful transition metal ions in the cellular environment (Eteshola et al., 2020). Indeed, one of the up-regulated functions in our study is a transferrin-initiating factor (see Supplementary Table S3 which lists DEGs supporting the most significant enriched GO terms). The GO term “mitochondrion” was not significant at warm temperature hypoxic exposure.

#### 3.2.2 *Functional response of South American* Aequiyoldia *to cooling and hypoxia*

In SSA, enriched GO terms in response to hypoxia were almost all linked to the regulation of apoptosis, e.g., programmed cell death, protein ubiquitination for proteasomal digestion, under *in situ* and low temperature conditions. The same apoptotic signal was observed when South American *A. cf. eightsii* were exposed to the combination of warmer *in situ* temperature and hypoxia (T_is_Ox_hyp_) compared to animals exposed to cooling and normoxia (T_c_Ox_n_). By comparison, the absence of apoptotic GO terms under hypoxic exposure in Antarctic *A. cf. eightsii* reveals a greater tolerance to oxygen depletion at *in situ* temperature and moderate warming, presumably supported by mitochondrial uncoupling *via* alternative oxidase and control of tissue damage through transition metal binding (McDonald and Gospodaryov, 2019; Eteshola et al., 2020).

**FIGURE 4:**
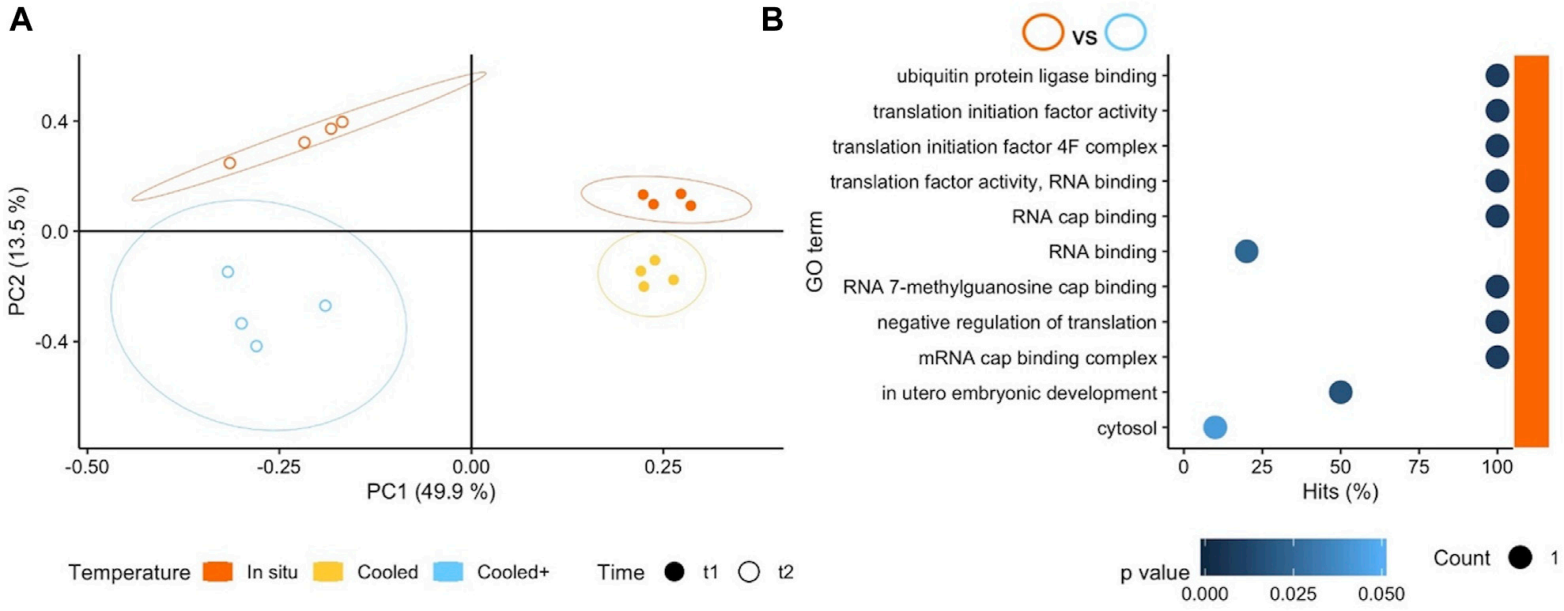
**(A)** Principal Component Analysis based on counts of differentially expressed genes (DEGs) for all three exposure temperatures applied in southern South America (SSA). Genes included in the analysis were significant (*p* < 0.001) in at least one pairwise comparison. Colours and shapes identify temperature and time of collection, respectively, and a 95% confidence ellipse is shown for each of the four treatments. **(B)** GO enrichment analysis of pairwise comparisons between T_c+_Ox_n_ (2°C) and T_is_Ox_n_ (7°C) at t_2_. The *x axis* indicates the percentage of DEGs found in a certain category over the total number of genes in the category (Hits). Size code indicates the number of DEGs in the GO term category (Count) and a gradient of colour indicates the *p*-value of the over represented category. Note that only the treatment T_is_Ox_n_ (7°C) indicated with colour on the right of the plot resulted in enriched terms.

We also tested the response of South American *A. cf. eightsii* to stepwise cooling to Antarctic shallow water summer temperatures, 2 °C (T_c+_) without additional hypoxic stress. Results in terms of numbers of DEGs between T_c+_Ox_n_ (2°C) and T_is_Ox_n_ (7°C) at t_2_ did not differ from those observed between T_c_Ox_n_ (4°C) and T_is_Ox_n_ (7°C) at t_1_ (15 and 14 DEGs, respectively). This indicates that bivalves from SSA can physiologically tolerate 21 days of exposure to current and future Antarctic summer temperatures under normoxic conditions*.* However, the differential gene expression pattern at t_2_ was more heterogeneous between and within treatments than at t_1_ (Figure 4A), and the small number of DEGs resulted in the enrichment of rather distinct GO terms between both comparisons and time points. While at t_1_ (4°C *vs*. 7°C, 10 days) various, not clearly interconnected functions were enriched (see Supplementary Table S2), the comparison at t_2_ (2°C *vs*. 7°C, 21 days) resulted exclusively in enriched GO terms at the *in situ* temperature. Most of these terms were based on only single DEGs and related to RNA binding and translation (Figure 4B). The enrichment of GO terms as RNA binding and translation at the higher *in situ* temperature suggests a lowered protein synthesis/turnover at the low temperatures awaiting SSA *Aequiyoldia* crossing the Drake.

## 4 Discussion

In this work, we analysed the transcriptomic response of two *Aequiyoldia* sibling lineages from SSA and WAP to exposure scenarios simulating a crossing of the Drake Passage, and to an expectable near future warming scenario at the WAP. We showed that the exposure to relatively small temperature changes and hypoxia, predicted to occur at higher frequency in shallow water environments at SSA and WAP, act as combined stressors (Sampaio et al., 2021) in *Aequiyoldia cf. eightsii*. Interestingly, the effects of hypoxia in both lineages surpassed those imposed in an upcoming warming-only scenario forecasted for the WAP, and those caused by a thermal transition in a hypothetical cross-continental invasion, with functional responses differing between *A. cf. eightsii* lineages. The magnitude of the response (number of DEGs) was greater under combined hypoxia and elevated temperature, highlighting the threat from the synergistic impact of both parameters in a global warming context. The relatively moderate response to warming as a single stressor of Antarctic *A. cf. eightsii* is noteworthy, as Antarctic invertebrates are usually expected to be highly sensitive to even small thermal increments (Peck et al., 2010). In contrast, cooling down SSA specimens to summer temperatures already occurring in WAP nearshore environments today (2°C), or expected in the near future if warming continues (4°C), did not lead to a significant change in the gene expression of South American bivalves. This could indicate that water temperature *per se* is unlikely to have formed an effective physiological barrier that prevents migration of South American *A. cf. eightsii* to the WAP. However, it should not be lost sight of the fact that the duration of our experiment and the focus on a single development stage (adults) does not allow us to hypothesize about the possibility of a permanent establishment of specimens in Antarctica and a successful life cycle.

### 4.1 Physiological response to thermal shifts and potential cross-continental invasions

In both lineages, a moderate shift in temperature by itself (without exposure to hypoxia) resulted in a small number of DEG, compared to what was found between oxygenic conditions, and we observed few clearly supported functional adjustments in response to cooling South American or warming Antarctic *A. cf. eightsii*.

The small effect of temperature (cooling) in the South American *A. cf. eightsii* is not surprising. South American *A. cf. eightsii* inhabit both subtidal and intertidal zones in Magellan Strait where annual temperature windows range from 5.9°C to 9.9°C in sublittoral and from 1.5°C to 18°C in shallow intertidal waters (Cardenas et al., 2020); a range that covers the acclimation temperatures of our cooling experiment and overlaps with current WAP water temperatures. For South American *A. cf. eightsii* 2°C is, however, a temperature minimum they will experience in the natural environment only rarely and not for prolonged periods of time. The functional analysis indicates lower rates of gene transcription and protein translation, as well as protein degradation (ubiquitination) in bivalves from SSA maintained under cooling conditions (2°C) when compared to the *in situ* temperature group (7°C). Slow-down of tissue growth and protein turnover are typically observed in marine invertebrates at the lower end of their thermal tolerance spectrum, and support energetic homeostasis during seasonal cold exposure (Fraser and Rogers, 2007). Lower efficiency of translation and protein synthesis at low temperatures are well-documented phenomena in marine invertebrates and fish (Fraser et al., 2002; Storch et al., 2005) from different climatic zones. Under the current summer temperature conditions at the northern WAP, a successful invasion by South American *A. cf eightsii* may still be thermally constrained, a hindrance that likely becomes less effective as future Antarctic summer temperatures will rise more frequently above 2°C. Interestingly, the functional analysis did not indicate upregulation of anaerobic metabolism or stress signals in cold-exposed *Aequiyoldia* from SSA and thus suggesting no acute energetic shortage. As the functional analysis did not reveal the same difference in protein synthesis and turnover capacities upon acclimation to 4°C (comparison T_c_Ox_n_: 4°C *vs*. T_is_Ox_n_: 7°C), successful colonization of Antarctic shallows by South American bivalves appears even more likely once these higher temperatures are reached more frequently in the WAP.

Moderate warming of the Antarctic *A. cf. eightsii* caused a slightly stronger overall response (90 DEGs in WAP *vs*. 14 DEGs in SSA) which might suggest higher susceptibility, but also adaptive competence toward moderate thermal challenge. Upregulation of immune responsive genes accounts for faster growth of bacteria and viruses under warmer conditions, especially in an experimental set-up. It shows that the bivalves were able to enhance immune competence under mild warming and thus respond to one of the major ecological complications in a climate change scenario (Mackenzie et al., 2014). Notably, both congeneric lineages failed to exhibit other typical responses to thermal stress such as the activation of heat shock proteins (HSP) which is commonly observed in many species, including bivalves, under exposure to a wide range of environmental stressors (Kim et al., 2017; Navarro et al., 2020). The absence of a heat shock response (HSR) in our GO analysis is, however, not completely unexpected. An inducible HSR is often missing (Clark and Peck, 2009; Clark et al., 2016), or not very pronounced (Koenigstein et al., 2013) in Antarctic marine invertebrates. It is likely that both lineages have a constitutive expression of heat shock genes sufficient to withstand the thermal stress to which they were exposed in the experiment. Likewise, we found no indication for an oxidative stress response, e.g., upregulation of antioxidant enzymes or metal chaperones under mild thermal challenge without hypoxic stress. In view of the physiological response to warming, as the decrease in antioxidant response at 5°C (Abele et al., 2001) and the results of the current transcriptome analysis (relatively high number of DEGs involving immune responses), 4°C could be the current threshold beyond which temperature in itself results damaging for Antarctic *A. cf. eightsii*. This clearly puts a limit to potential northward invasions under the present conditions.

### 4.2 The functional response to hypoxia differs between both lineages of *Aequiyoldia*

Exposure to hypoxia (10 days at <5% O_2_, Figure 1) had more pronounced effects on *A cf. eightsii* gene expression than moderate warming/cooling alone on both sides of Drake Passage (numbers of DEGs). Thus, hypoxia represents a severe challenge, irrespective of the actual temperature change, cooling or warming in SSA as well as WAP. The dominance of apoptosis-related functions, which occurred only in South American *Aequiyoldia* bivalves, emphasizes a greater susceptibility to oxygen deficiency under the conditions prevailing in SSA. The regulation of apoptosis has been suggested to be an emergency mechanism of tolerance under acute oxygenation stress mainly in organisms adapted to frequent oxygen fluctuations (Falfushynska et al., 2020). Apoptosis mitigates tissue inflammation by reducing prevalence of uncontrolled cell death (necrosis), but this mitigation strategy comes at a high physiological cost and inevitably leads to cell loss and functional impairment; so, the extent of tissue apoptosis correlates negatively with the survival of organisms under hypoxia (Falfushynska et al., 2020).

On the other hand, the response to hypoxia of Antarctic *Aequiyoldia* involved the regulation of genes supporting mitochondrial adjustments including the expression of the Alternative oxidase (AOX) under *in situ* temperature (Figure 2A; Supplementary Table S3). The AOX reduces oxygen to water using electrons from the ubiquinol pool when cytochrome oxidase is inhibited by nitric oxide (Strahl and Abele, 2020) or sulfide (Völkel and Grieshaber, 1996). This alternative electron pathway forms a shortcut of the “modern” (canonical) mitochondrial ETS and causes uncoupling of complexes III and IV. By doing so, it reduces the efficiency of mitochondrial ATP production and flattens mitochondrial inner membrane potential in a state of transient metabolic rate depression. The suggested advantage of this mitigation strategy lies in avoiding an oxidative burst response and the associated excess production of damaging ROS during frequent hypoxia-reoxygenation cycling, typical for marine sedimentary habitats (Abele et al., 2017; McDonald and Gospodaryov, 2019). Indeed, genes encoding for AOX are mostly inherited in hypoxia-tolerant marine invertebrates ranging from sponges and cnidarians to ascidians, including marine and freshwater bivalves (McDonald et al., 2009; Jacobs and Ballard, 2022). Inducible expression of AOX has been reported in oysters exposed to hypoxia-reoxygenation (Sussarellu et al., 2012) as well as in hypoxia-exposed freshwater bivalves from Andean lakes (Yusseppone et al., 2018). To the best of our knowledge, this is the first time that AOX upregulation is reported for an Antarctic metazoan. The expression of AOX might explain the ecological success of *A. cf. eightsii* in coastal and fjordic systems such as Potter Cove, where sedimentary conditions vary at local and temporal scale. Sedimentary redox profiles can vary from a few centimetres of oxic surface layer in glacial vicinity where organic carbon content is low, to sub- and anoxic conditions in areas where debris from macroalgal belts is deposited and microbially recycled. Despite this pronounced and unpredictable temporal and spatial variation in oxygen availability *A. cf. eightsii* is an abundant infaunal component (Monien et al., 2014; Pasotti et al., 2015).

The response to hypoxia of the Antarctic *A. cf. eightsii* under warming condition did not involve this protective mitochondrial adjustment (Figure 2B). Instead, it caused a stress response with enhanced metal binding either to reduce oxidative stress originating from cellular transition metal pools (Eteshola et al., 2020), or to increase the capacity of oxygen binding to intracellular haemoglobin in gills or to hemocyanin in the hemolymphatic fluid of *A. cf. eightsii* (Angelini et al., 1998).

Lower environmental temperatures are known to alleviate the hypoxic stress in marine invertebrates by reducing their metabolic requirements, especially if they can enter a state of suspended animation. Hence, although the TOC:TS ratios indicated similar oxygenation conditions in the sediment at both collection sites (suboxic, Supplementary Table S1), at present Antarctic specimens still have better chances of survival. It must also not be forgotten that human influence on the coasts in South America (including cities, livestock, agriculture and in particular the rapidly expanding salmon farming) is expected to promote a higher variability in oxygen conditions (Levin et al., 2009; Quinones et al., 2019), and that pollution can have additional adverse effects that endanger sedimentary fauna locally.

## 5 Conclusion

Our results suggest that a physiological component that has previously prevented a successful transition of South American bivalves to Antarctica is likely to exist and that sustained warming of the WAP would thus increase the invasibility of the Antarctic benthos by species currently residing in South America. The reverse direction of successful long-distance dispersal (i.e., from the Antarctic to South America), while not completely impossible, is probably less likely as far as physiology as a contributing factor is concerned. By the time the warming on the WAP would have lasted long enough to yield adaptations that would increase the likelihood of survival under temperatures prevailing in South America nowadays, the temperatures there, too, would have increased sufficiently to make establishing a successful foothold of Antarctic bivalves north of the Drake physiologically as difficult as it is already now. In Antarctica, *A. cf. eightsii* appear to have some capacity to withstand exposure to hypoxia as an added stressor, e.g., by switching to the alternative oxidase pathway. The induction of AOX in Antarctic *Aequiyoldia* under hypoxia and *in situ* temperature is remarkable, and the rearrangement of the mitochondrial electron transport system may indeed be central for bivalves’ hypoxia endurance and more generally for their reported ecological success. The high prevalence of cell death-related functions under hypoxia in South American *A. cf. eightsii* might suggest that this species may not be amongst the winners of sustained warming there. While it is possible that a temperature rise will alter the composition of faunal communities in SSA, too, our results suggest that the winners of such future change will be recruited from the pool of species already populating the shores of South America as they would probably be already adapted to more variable conditions and having a smaller temperature differential to contend with compared to possible invasive species coming across the Drake from WAP.

## Data Availability

The datasets presented in this study can be found in online repositories. The names of the repository/repositories and accession number(s) can be found below: https://www.ebi.ac.uk/ena, ERR4265443, ERR4276392—ERR4276460.
