# Supplementary material for: A molecular perspective on the invasibility of the southern ocean benthos: The impact of hypoxia and temperature on gene expression in South American and Antarctic Aequiyoldia bivalves
Source: Front Physiol. 2023 Feb 21;14:1083240. doi: 10.3389/fphys.2023.1083240 (PMC9989211; doi:10.3389/fphys.2023.1083240)

Supplementary Material

# Supplementary Tables

**Supplementary Table 1**

Environmental data registered at the time of animal collection in West Antarctic Peninsula (WAP) and Southern South America (SSA): depth, bottom salinity and temperature, total organic carbon (TOC) and total sulphur (TS) of sediment, and TOC: TS ratio.

|  | Depth (m) | Salinity | Temperature (°C) | TOC (%) | TS (%) | TOC: TS |
| --- | --- | --- | --- | --- | --- | --- |
| WAP | 5.88 | 33.80 | 1.67 | 0.79 | 0.25 | 3.16 |
| SSA | 1.50 | 32.00 | 7.00 | 0.95 | 0.27 | 3.52 |

**Supplementary Table 2**

Complete list of GO enriched terms resulting from each pairwise comparisons in West Antarctic Peninsula (WAP) and southern South America (SSA) experiments. The first column indicates the treatment in which the corresponding GO term resulted enriched. The column ‘numDEInCat’ indicates the number of differentially expressed genes in the category.

| **WAP experiment** |  |  |  |
| --- | --- | --- | --- |
|  |  |  |  |
| **T_in_Ox_hip_ vs. T_in_Ox_n_** |  |  |  |
| Enrich. in treatment: | category | numDEInCat | term |
| T_in_Ox_hip_ | GO:0005739 | 4 | mitochondrion |
| T_in_Ox_hip_ | GO:0006357 | 3 | regulation of transcription by RNA polymerase II |
| T_in_Ox_hip_ | GO:0042025 | 3 | host cell nucleus |
| T_in_Ox_n_ | GO:0005856 | 1 | cytoskeleton |
|  |  |  |  |
| **T_in_Ox_hip_ vs. T_w_Ox_hip_** |  |  |  |
| Enrich. in treatment: | category | numDEInCat | term |
| T_in_Ox_hip_ | GO:0046982 | 2 | protein heterodimerization activity |
| T_in_Ox_hip_ | GO:0003197 | 1 | endocardial cushion development |
| T_in_Ox_hip_ | GO:0007162 | 1 | negative regulation of cell adhesion |
| T_in_Ox_hip_ | GO:0007422 | 1 | peripheral nervous system development |
| T_in_Ox_hip_ | GO:0009968 | 1 | negative regulation of signal transduction |
| T_in_Ox_hip_ | GO:0014037 | 1 | Schwann cell differentiation |
| T_in_Ox_hip_ | GO:0014065 | 1 | phosphatidylinositol 3-kinase signaling |
| T_in_Ox_hip_ | GO:0016328 | 1 | lateral plasma membrane |
| T_in_Ox_hip_ | GO:0019838 | 1 | growth factor binding |
| T_in_Ox_hip_ | GO:0021545 | 1 | cranial nerve development |
| T_w_Ox_hip_ | GO:0007160 | 2 | cell-matrix adhesion |
| T_w_Ox_hip_ | GO:0005112 | 1 | Notch binding |
| T_w_Ox_hip_ | GO:0005576 | 2 | extracellular region |
| T_w_Ox_hip_ | GO:0003180 | 1 | aortic valve morphogenesis |
| T_w_Ox_hip_ | GO:0005581 | 1 | collagen trimer |
| T_w_Ox_hip_ | GO:0010629 | 1 | negative regulation of gene expression |
| T_w_Ox_hip_ | GO:0010811 | 1 | positive regulation of cell-substrate adhesion |
| T_w_Ox_hip_ | GO:0032966 | 1 | negative regulation of collagen biosynthetic process |
| T_w_Ox_hip_ | GO:0034668 | 1 | integrin alpha4-beta1 complex |
| T_w_Ox_hip_ | GO:0050866 | 1 | negative regulation of cell activation |
|  |  |  |  |
| **T_in_Ox_n_ vs. T_w_Ox_n_** |  |  |  |
| Enrich. in treatment: | category | numDEInCat | term |
| T_w_Ox_n_ | GO:0002281 | 1 | macrophage activation involved in immune response |
| T_w_Ox_n_ | GO:0003823 | 1 | antigen binding |
| T_w_Ox_n_ | GO:0030277 | 1 | maintenance of gastrointestinal epithelium |
| T_w_Ox_n_ | GO:0030299 | 1 | intestinal cholesterol absorption |
| T_w_Ox_n_ | GO:0030308 | 1 | negative regulation of cell growth |
| T_w_Ox_n_ | GO:0042632 | 1 | cholesterol homeostasis |
| T_w_Ox_n_ | GO:0046790 | 1 | virion binding |
| T_w_Ox_n_ | GO:0099512 | 1 | supramolecular fiber |
| T_w_Ox_n_ | GO:0043231 | 1 | intracellular membrane-bounded organelle |
|  |  |  |  |
| **T_w_Ox_hip_ vs. T_w_Ox_n_** |  |  |  |
| Enrich. in treatment: | category | numDEInCat | term |
| T_w_Ox_hip_ | GO:0046872 | 5 | metal ion binding |
| T_w_Ox_hip_ | GO:0004222 | 2 | metalloendopeptidase activity |
| T_w_Ox_hip_ | GO:0003677 | 4 | DNA binding |
| T_w_Ox_n_ | GO:0000977 | 1 | RNA polymerase II regulatory region DNA binding |
| T_w_Ox_n_ | GO:0002376 | 1 | immune system process |
| T_w_Ox_n_ | GO:0005925 | 1 | focal adhesion |
| T_w_Ox_n_ | GO:0006355 | 1 | regulation of transcription, DNA-templated |
| T_w_Ox_n_ | GO:0007596 | 1 | blood coagulation |
| T_w_Ox_n_ | GO:0051607 | 1 | defense response to virus |
| T_w_Ox_n_ | GO:0060333 | 1 | interferon-gamma-mediated signaling pathway |
| T_w_Ox_n_ | GO:0060337 | 1 | type I interferon signaling pathway |
|  |  |  |  |
| **SSA experiment** |  |  |  |
|  |  |  |  |
| **T_c_Ox_hip_ vs. T_c_Ox_n_** |  |  |  |
| Enrich. in treatment: | category | numDEInCat | term |
| T_c_Ox_hip_ | GO:0000122 | 4 | negative regulation of transcription by RNA polymerase II |
| T_c_Ox_hip_ | GO:0031398 | 3 | positive regulation of protein ubiquitination |
| T_c_Ox_hip_ | GO:0043027 | 3 | endopeptidase inhibitor activity involved in apoptotic process |
| T_c_Ox_hip_ | GO:0043154 | 3 | negative regulation of endopeptidase involved in apoptotic process |
| T_c_Ox_hip_ | GO:0061630 | 3 | ubiquitin protein ligase activity |
| T_c_Ox_hip_ | GO:1990001 | 3 | inhibition of endopeptidase activity involved in apoptotic process |
| T_c_Ox_hip_ | GO:0043066 | 4 | negative regulation of apoptotic process |
| T_c_Ox_hip_ | GO:0005634 | 8 | nucleus |
|  |  |  |  |
| **T_in_Ox_n_ vs. T_c_Ox_hip_** |  |  |  |
| Enrich. in treatment: | category | numDEInCat | term |
| T_c_Ox_hip_ | GO:0045944 | 4 | positive regulation of transcription by RNA polymerase II |
| T_c_Ox_hip_ | GO:0000122 | 4 | negative regulation of transcription by RNA polymerase II |
| T_c_Ox_hip_ | GO:0005667 | 3 | transcription factor complex |
| T_in_Ox_n_ | GO:0016021 | 3 | integral component of membrane |
|  |  |  |  |
| **T_in_Ox_h_ vs. T_c_Ox_n_** |  |  |  |
| Enrich. in treatment: | category | numDEInCat | term |
| T_c_Ox_n_ | GO:0006313 | 2 | transposition, DNA-mediated |
| T_c_Ox_n_ | GO:0016324 | 2 | apical plasma membrane |
| T_in_Ox_h_ | GO:0031398 | 3 | positive regulation of protein ubiquitination |
| T_in_Ox_h_ | GO:0043027 | 3 | endopeptidase inhibitor activity involved in apoptotic process |
| T_in_Ox_h_ | GO:0043154 | 3 | negative regulation of endopeptidase involved in apoptotic process |
| T_in_Ox_h_ | GO:0061630 | 3 | ubiquitin protein ligase activity |
| T_in_Ox_h_ | GO:1990001 | 3 | inhibition of endopeptidase involved in apoptotic process |
| T_in_Ox_h_ | GO:0019899 | 3 | enzyme binding |
|  |  |  |  |
| **T_in_Ox_n_ vs. T_c_Ox_n_** |  |  |  |
| Enrich. in treatment: | category | numDEInCat | term |
| T_c_Ox_n_ | GO:0001674 | 1 | female germ cell nucleus |
| T_c_Ox_n_ | GO:0005534 | 1 | galactose binding |
| T_c_Ox_n_ | GO:0005623 | 1 | cell |
| T_c_Ox_n_ | GO:0033296 | 1 | rhamnose binding |
| T_c_Ox_n_ | GO:0042564 | 1 | NLS-dependent protein nuclear import complex |
| T_c_Ox_n_ | GO:0060473 | 1 | cortical granule |
| T_c_Ox_n_ | GO:1903777 | 1 | melibiose binding |
| T_c_Ox_n_ | GO:0042803 | 1 | protein homodimerization activity |
| T_c_Ox_n_ | GO:0005524 | 1 | ATP binding |
| T_in_Ox_n_ | GO:0005506 | 1 | iron ion binding |
| T_in_Ox_n_ | GO:0045329 | 1 | carnitine biosynthetic process |
|  |  |  |  |
| **T_in_Ox_n_ vs. T_c_Ox_hip_** |  |  |  |
| Enrich. in treatment: | category | numDEInCat | term |
| T_c_Ox_hip_ | GO:0031398 | 3 | positive regulation of protein ubiquitination |
| T_c_Ox_hip_ | GO:0043027 | 3 | endopeptidase inhibitor activity involved in apoptotic process |
| T_c_Ox_hip_ | GO:0043154 | 3 | negative regulation of endopeptidase involved in apoptotic process |
| T_c_Ox_hip_ | GO:0061630 | 3 | ubiquitin protein ligase activity |
| T_c_Ox_hip_ | GO:1990001 | 3 | inhibition of endopeptidase activity involved in apoptotic process |
| T_c_Ox_hip_ | GO:0043066 | 4 | negative regulation of apoptotic process |
| T_in_Ox_n_ | GO:0003924 | 2 | GTPase activity |

**Supplementary Table 3**

List of differentially expressed genes (DEG) supporting the most significant enriched GO terms in hypoxia vs. Normoxia treatments at in situ and warming conditions in the experiment performed in West Antarctic Peninsula

| **T_is_Ox_n_ vs. T_is_Ox_hyp_**  GO: Mitochondrion |
| --- |
| ETS domain-containing protein Elk-1 |
| Haloacid dehalogenase-like hydrolase domain-containing 5 |
| Alternative oxidase, mitochondrial |
| AAC-rich mRNA clone AAC4 protein |
|  |
| **T_w_Ox_n_ vs. T_w_Ox_hyp_**  GO: metal ion binding |
| Elastase |
| Cytochrome b |
| Transcription initiation factor TFIID subunit 3 |
| Neprilysin-1 |
|  |
| GO: metal ion binding |
| Elastase |
| Neprilysin-1 |

# Supplementary Figures

**Supplementary Figure 1**

Map showing the regions where the experiments were conducted: Southern South America (SSA) and West Antarctic Peninsula (WAP).

**
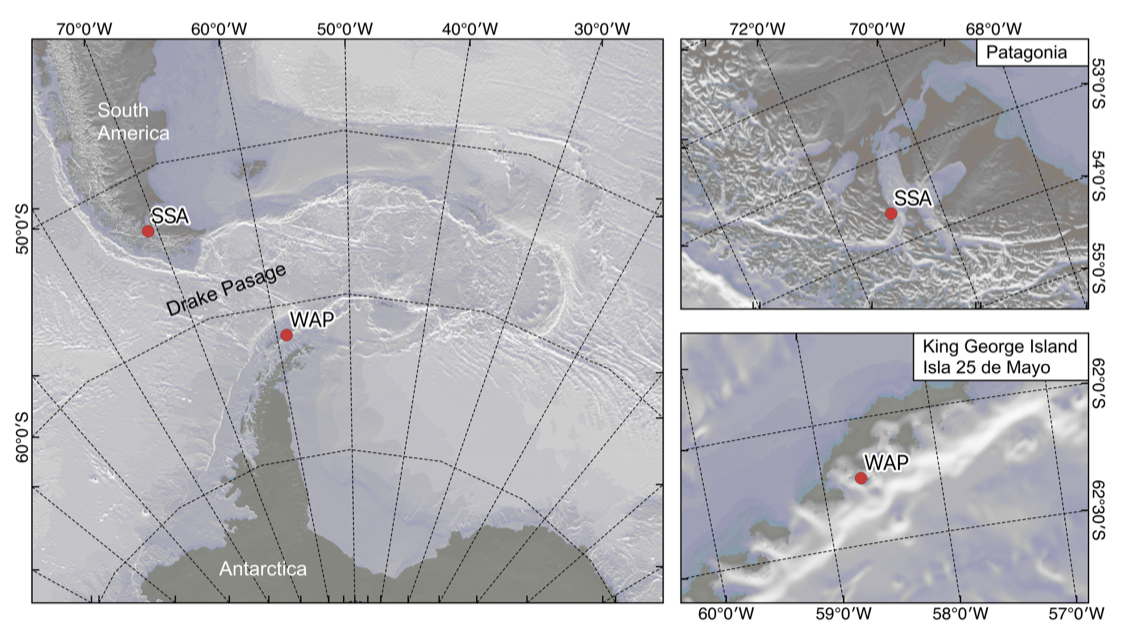
**

**Supplementary Figure 2**

Experimental design carried out in Southern South America (SSA) and West Antarctic Peninsula (WAP). In both regions the experimental organisms were acclimatized for 10 days. The experimental phase involved the exposure of 4 replicates (jars) containing five animals for 18 days to the following conditions: in SSA: i*n situ* (7 °C) and South migration future scenario (4 °C) in combination with normoxia and hypoxia; in WAP: *in situ* (1.5 °C) and warming (4 °C) in combination with normoxia and hypoxia. In the experiment in SSA, an additional step was conducted on day 18, by further decreasing the temperature from 4 °C down to 2 °C under conditions of normoxia.


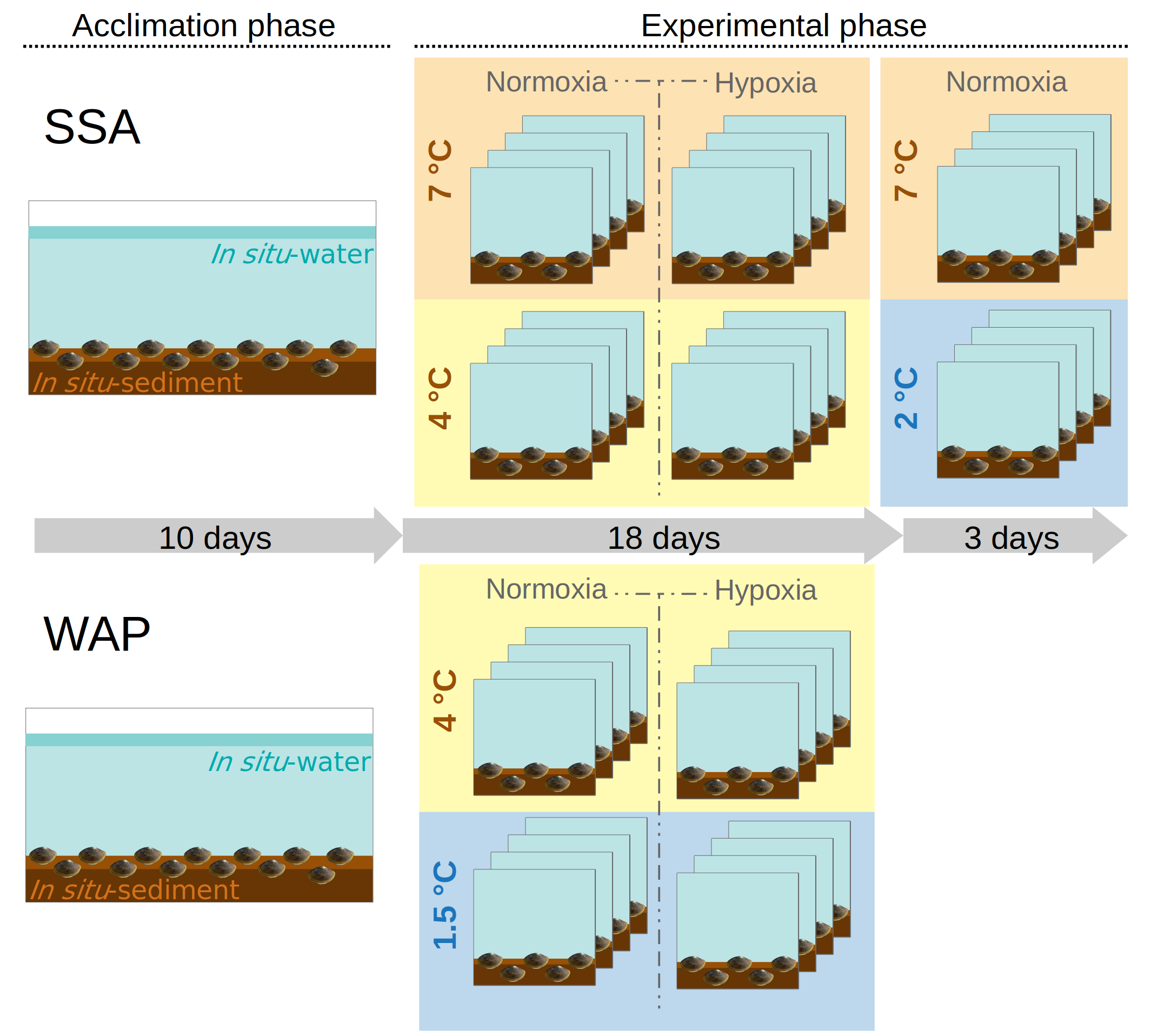

Supplement: Supplementary file 1 [file DataSheet1.docx]
